# Downregulation of endometrial mesenchymal marker SUSD2 causes cell senescence and cell death in endometrial carcinoma cells

**DOI:** 10.1371/journal.pone.0183681

**Published:** 2017-08-25

**Authors:** Shaqiu Zhang, Ni Zeng, Nour Alowayed, Yogesh Singh, Anchun Cheng, Florian Lang, Madhuri S. Salker

**Affiliations:** 1 Institute of Preventive Veterinary Medicine, Sichuan Agricultural University, Wenjiang, Chengdu, Sichuan, P.R. China; 2 Department of Internal Medicine III, Tübingen University, Tübingen, Germany; 3 Institute of Medical Genetics and Applied Genomics, Tübingen University, Tübingen, Germany; 4 Department of Physiology I, Tübingen University, Tübingen, Germany; 5 Research Institute for Women's Health, University Hospital Tübingen, Tübingen, Germany; National Cancer Institute, UNITED STATES

## Abstract

The cause of death among the majority of endometrial cancer patients involves migration of cancer cells within the peritoneal cavity and subsequent implantation of cancer spheroids into neighbouring organs. It is, thereby, important to identify factors that mediate metastasis. Cell adhesion and migration are modified by the mesenchymal stem cell (MSC) marker Sushi domain containing 2 (SUSD2), a type I transmembrane protein that participates in the orchestration of cell adhesion and migration through interaction with its partner Galactosidase-binding soluble-1 (LGALS1). MSCs have emerged as attractive targets in cancer therapy. Human endometrial adenocarcinoma (Ishikawa) cells were treated with TGFβ (10 ng/ml) for 72h. *SUSD2*, *LGALS1* and *MKI67* transcript levels were quantified using qRT-PCR. The proportion of SUSD2 positive (SUSD2+) cells and SMAD2/3 abundance were quantified by FACS and Western blotting, respectively. Senescent cells were identified with β-galactosidase staining; cell cycle and cell death were quantified using Propidium Iodide staining. Treatment of endometrial cancer cells (Ishikawa cells) with TGFβ (10 ng/ml) significantly decreased *SUSD2* transcript levels and the proportion of SUSD2 positive cells. Silencing of *SUSD2* using siRNA resulted in senescence and cell death of Ishikawa cells *via* activation of SMAD2/3. These findings suggest that SUSD2 counteracts senescence and cell death and is thus a potential chemotherapeutic target in human endometrial cancer.

## Introduction

Endometrial cancer is the most common gynaecological malignancy, the incidence of which is increasing worldwide [1]. Endometrial cancers are often classified into two general clinicopathological types. Type I endometrial tumors, which account for ~70% of endometrial cancers, are primarily comprised of low-grade endometrioid tumors and are associated with favorable prognosis. Type II endometrial cancers comprise a group of high-risk tumors of serous, clear cell or high-grade endometrioid histology that are highly invasive and associated with poor survival [2]. Approximately 75% of all endometrial cancer related deaths can be attributed to the aggressive behaviour of these high-risk tumours [2]. Investigations on the molecular mechanisms contributing to endometrial cancer metastasis could lead to the development of improved therapeutic strategies.

Mesenchymal stem cells (MSCs) are characterized by the expression of cell surface markers such as CD73, CD90, and CD105, and the absence of expression of hematopoietic lineage markers [3]. MSCs can differentiate into several mesenchymal lineages such as osteoblasts, adipocytes, chondrocytes and potentially other skeletal tissue cells by culturing MSCs under defined mechano-chemical conditions [4]. MSCs contribute to carcinogenesis [4], as they can home to tumour sites and contribute to tumour growth and cancer progression [5]. The high proliferative capacity and regenerative potential are the main phenotypes of MSCs [6]. MSCs proliferation and dedifferentiation is inhibited by TGFβ [7], TGFβ signalling is initiated by ligand-induced oligomerization of serine/threonine receptor kinases and phosphorylation of the cytoplasmic signalling molecules Smad2 and Smad3 [8]. Carboxy-terminal phosphorylation of Smads by activated receptors results in their partnering with the common signalling transducer Smad4, and translocation to the nucleus [8, 9]. Activated Smads regulate diverse biological effects in concert with other transcription factors resulting in cell-state specific modulation of transcription or epigenetic modifications and senescence [10, 11]. Cellular senescence results in a permanent cell cycle arrest and loss of the self-renewal potential [12]. TGFβ has been demonstrated to induce senescence of tumour cells and other cell lines [13–16]. The effect of TGFβ and/or SMADs on senescence of endometrial cancer cells has not yet been elucidated.

MSCs bind to W5C5, an antibody that recognizes Sushi Domain containing 2 (SUSD2) and has thus been introduced as a marker for the prospective isolation of endometrial MSCs [17–19]. MSCs and SUSD2, have been identified in the endometrium [20, 21]. SUSD2 is a type I transmembrane protein of 820 amino acids consisting of a large extracellular region containing a Somatomedin B (SMB), an adhesion associated domain in mucin 4 (MUC4) and other Proteins (AMOP), a Von Willebrand factor (vWF), and a Sushi domain [22]. AMOP domains are considered to be involved in cell adhesion and represent an important component of MUC4, the most related protein to SUSD2 [23, 24]. Proteins containing vWF domains participate in numerous biological events including cell adhesion, migration, homing and signal transduction, and are involved in interactions with a large array of ligands [25]. Sushi domains, also known as Complement Control Proteins (CCP) module or Short Consensus Repeats (SCR), are components of a variety of complement and adhesion proteins [26].

SUSD2 is overexpressed in ovarian cancer as well as in several brain cancers, including ependymoma, glioblastoma, and medulloblastoma [27, 28]. SUSD2 binds to its interaction partner Galectin-1 (LGALS1). It has been shown that TGFβ can promote differentiation of SUSD2+ cells (purified benign endometrial MSCs) [29]. Furthermore, used of A83-01 (TGFβ receptor inhibitor) promoted SUSD2+ cell proliferation and blocked apoptosis *via* the SMAD 2/3 pathway [7]. Whether, the role of SUSD2 and TGFβ signalling *via* SMADs in endometrial cancer cells remained to be defined.

The present study explored whether the MSC marker SUSD2 is sensitive to TGFβ in human endometrial cancer (Ishikawa; well differentiated adenocarcinoma) cells. Surprisingly, we observed that TGFβ downregulates SUSD2 transcription and protein expression. Further studies revealed that loss of SUSD2 induces cell senescence and cell death *via* SMAD2/3.

## Materials and methods

### Cell culture

Cells of the well differentiated endometrial Ishikawa carcinoma cell line (#ECACC 99040201, Sigma, Germany) were cultured in DMEM/F12 without phenol red media (#21041–025, Life technologies, USA), containing 10% fetal bovine serum (#10270–106, life technologies), and 1% antibiotic/antimycotic solution (#P4333, Sigma) in a humidified atmosphere at 37°C and 5% CO_2_. Cells were treated as described with TGFβ (#14-8342-80, eBioscience, USA) for the indicated time points. Cells were routinely tested for mycoplasma.

### Knockdown of SUSD2

Ishikawa cells were transfected using DharmaFECT3 (#T-2003-01, Dharmacon, USA) as recommended by manufacturer’s guidelines using siRNA targeting-*SUSD2* (#L-010716-00–0005, Dharmacon) and control siRNA (#D-001910-01-05, Dharmacon).

### Quantitative Real-time PCR

Detection of gene expression was performed with KapaFast-SYBRGreen (#KAPBKK4606, Peqlab VWR, Germany) and quantitative RT-PCR was performed on a BioRad iCycler iQ^™^ Real-Time PCR Detection System (Bio-Rad Laboratories, Germany) as described earlier [30]. The expression levels of the samples were calculated by the 2^-ΔΔ^Ct method using *L19* to normalize for variances in input cDNA and expressed as arbitrary units. All measurements were performed in duplicate. Melting curve analysis and agarose gel electrophoresis confirmed amplification specificity. Primer sequences are available on request.

### Flow cytometry

Single-cell suspensions from cell cultures were acquired by trypsin-EDTA digestion (#25200–056, Life Technologies) and stained with human SUSD2-PE antibody (#327508; BioLegend, UK) then analyzed using a BD FACSCalibur™ (BD Bioscience, Germany). The data were analyzed by FlowJo software (FLOWJO, LLC, USA).

### Western blotting

Whole cell protein extracts were obtained by direct lysis in Lammelli’s Buffer and boiled at 100°C for 10 min. Proteins were separated on 12% SDS-polyacrylamide gels and transferred to PVDF membranes (#10600023, Amersham Biosciences, UK). Nonspecific binding sites were blocked for 1 hour at room temperature with 5% nonfat dry milk in TBS-T. Membranes were incubated overnight at 4°C with anti-SMAD2/3 (1:1000, #8685, Cell Signaling Technology, Germany) and anti-GAPDH (1:2000, #2118, Cell Signaling Technology). Membranes were washed 3 times with 1% TBS-T, followed by incubation with HRP-conjugated anti-rabbit secondary antibodies (1:1000, #7074, Cell Signaling Technology). Protein complexes were visualized with enhanced chemiluminescent HRP substrate (#R-03031-D25 and R-03025-D25, advansta, USA) and bands were quantified using ImageJ software (National Institute of Mental Health).

### Cell cycle analysis

After 72h treatment, cells and medium were collected into a 15 ml centrifuge tube and spun down at 600×*g* for 5 min. The supernatant was discarded and 1 ml of -20°C ice-cold ethanol (#20821.330, VWR Chemicals, USA): PBS (#D8537, Sigma) mixture (3:1) was added to the pellet during vortexing. The mixture was kept at -20°C overnight, the next day washed with PBS again, 250 μl PI mix containing 50 μg/ml PI (#P4864, Sigma) and 100 μg/ml RNase A (#R4642, Sigma) were added, incubated for 30 min at 37°C, and subjected to flow cytometry for cell cycle and death analysis. The data were analyzed by FlowJo software.

### Senescence associated β-galactosidase staining

Expression of pH-dependent senescence associated β-galactosidase (SA-β-gal) activity was analyzed simultaneously in control and SUSD2 knock down cells using the Senescence β-Galactosidase Staining Kit (#9860, Cell Signaling Technology) [31] following the manufacture’s guidelines. Briefly, cells were washed once with PBS, fixed for 15 minutes at room temperature in 1× fixative solution, washed again with PBS twice, and incubated at 37°C (without CO_2_) at least overnight with fresh β-Galactosidase Staining Solution. Cells were visualized by phase-contrast microscopy (Nikon Diaphot 300, Germany), data were acquired with Bresser Mikrocam (Bresser GmbH, Germany) camera using MikroCamLab software and analyzed with ImageJ software in triplicate, taking 4 random non-overlapping fields of view for each sample.

### Statistics

Data are provided as means ± SEM, *n* represents the number of independent experiments. All data were tested for significance using Student’s two-tailed t-test. Only results with *P*<0.05 were considered statistically significant.

## Results

**Fig 1:**
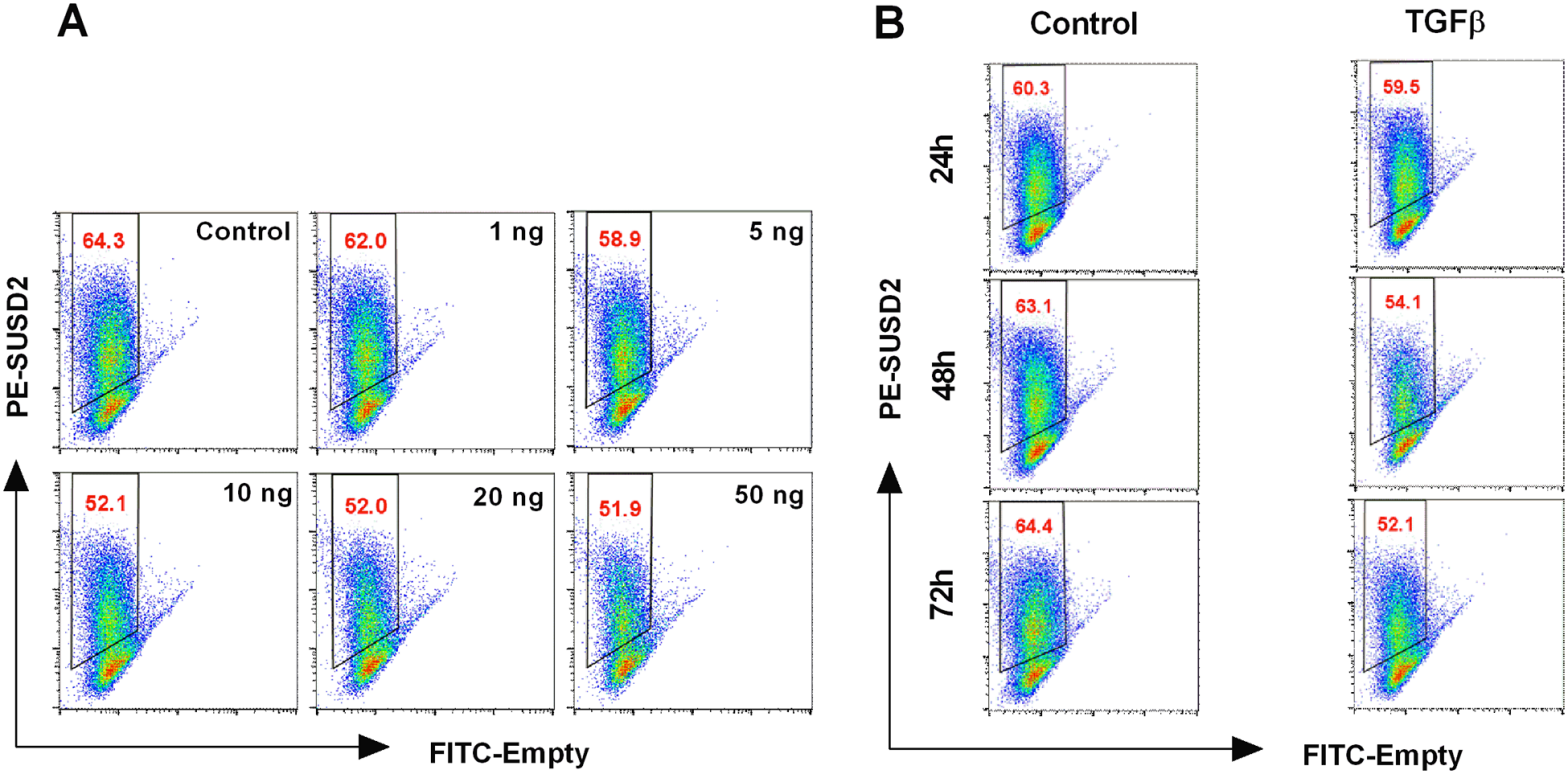
Titration and Time-course of the TGFβ effect on *SUSD2*+ Ishikawa cells. (A) Representative original FACS plots showing the effect of increasing TGFβ concentrations (Control, 1, 5, 10, 20, 50 ng/ml) for 72h on the percentage of SUSD2+ expressing cells. (B) Representative original FACS plots showing increasing abundance of SUSD2+ cells at 24h, 48h and 72h time points with TGFβ (10 ng/ml) or untreated (Control).

The present study addressed the effect of TGFβ on MSCs marker SUSD2 in Ishikawa cells, a well differentiated endometrial adenocarcinoma cell line. In a first series of experiments the proportion of SUSD2 positive (SUSD2+) cells was evaluated in dose-response and time-course experiments. To determine whether TGFβ affects SUSD2 levels, Ishikawa cell cultures were treated with 1, 5, 10, 20, or 50 ng/ml TGFβ for 72h. Cells were harvested and subjected to FACS. As seen in Fig 1A and S1A Fig, a treatment with 5 ng/ml TGFβ slightly but significantly decreased the number of SUSD2+ cells. At concentrations ranging from 10–50 ng/ml, TGFβ further reduced the percentage of SUSD2+ cells. To investigate the kinetics of TGFβ we performed time course studies. Ishikawa cells were treated with TGFβ (10 ng/ml) for a time course lasting up to 72h. As illustrated in Fig 1B and S1B Fig, at 72h TGFβ significantly decreased the number of SUSD2+ cells. The significant decrease of SUSD2 at protein level was paralleled by a similar decline of SUSD2 mRNA level (S1C Fig).

**Fig 2:**
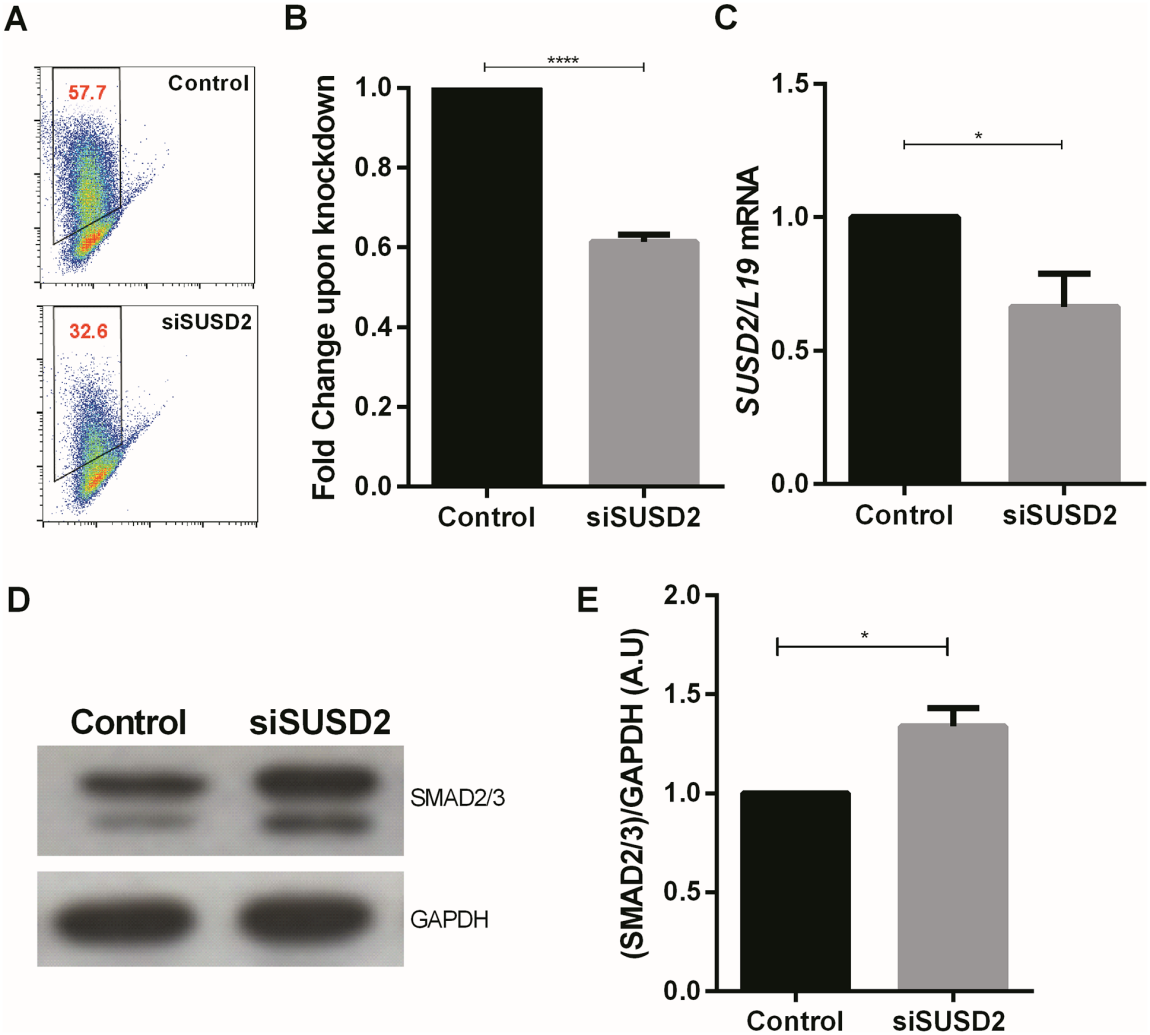
Effect of SUSD2 knockdown on SMAD2/3 expression. (A) Representative original FACS plots showing SUSD2 knockdown. (B) Arithmetic means ± SEM (n = 5) of SUSD2+ Ishikawa cells. Data are depicted as fold induction relative to non-targeting siRNA (Control) samples. ****P<0.0001 indicates statistically significant difference from control cells using Student’s t-test. (C) Arithmetic means ± SEM (n = 4) of *SUSD2* transcript levels normalized to *L19* transcript levels in Ishikawa cells. Data are depicted as fold induction relative to transcript levels of control samples. *P< 0.05 indicates statistically significant difference from control cells using Student’s t-test. (D) Representative original Western blot of SMAD2/3 and GAPDH in Ishikawa cells. (E) Arithmetic means ± SEM (n = 7) of SMAD2/3 ratio normalized to GAPDH in Ishikawa cells. *P< 0.05 indicates statistically significant difference from control cells using Student’s t-test.

TGFβ signals partially through activation of different SMAD family members [32]. Levels of both SMAD2/3 and SMAD1/5 are upregulated in cells undergoing senescence [33]. To investigate whether loss of SUSD2 affects SMAD proteins, Ishikawa cells were transfected with Non-Targeting (Control) oligos or SUSD2 siRNA. As illustrated in Fig 2A–2C the silencing was highly efficient. In parallel samples, we performed Western blotting to investigate the effect on SMAD2/3 proteins. To our surprise, levels of SMAD2/3 were significantly increased upon SUSD2 knockdown (Fig 2D & 2E). We next examined the expression of *MKi67* and *LGALS1* gene (the interaction partner for SUSD2). As a result, *LGALS1* was significantly reduced upon SUSD2 knockdown (S2A Fig). Moreover, *MKi67* encoding the proliferation marker Ki-67 antigen, was significantly attenuated upon SUSD2 knockdown, suggesting that indeed loss of SUSD2 is associated with decreased proliferation (S2B Fig).

**Fig 3:**
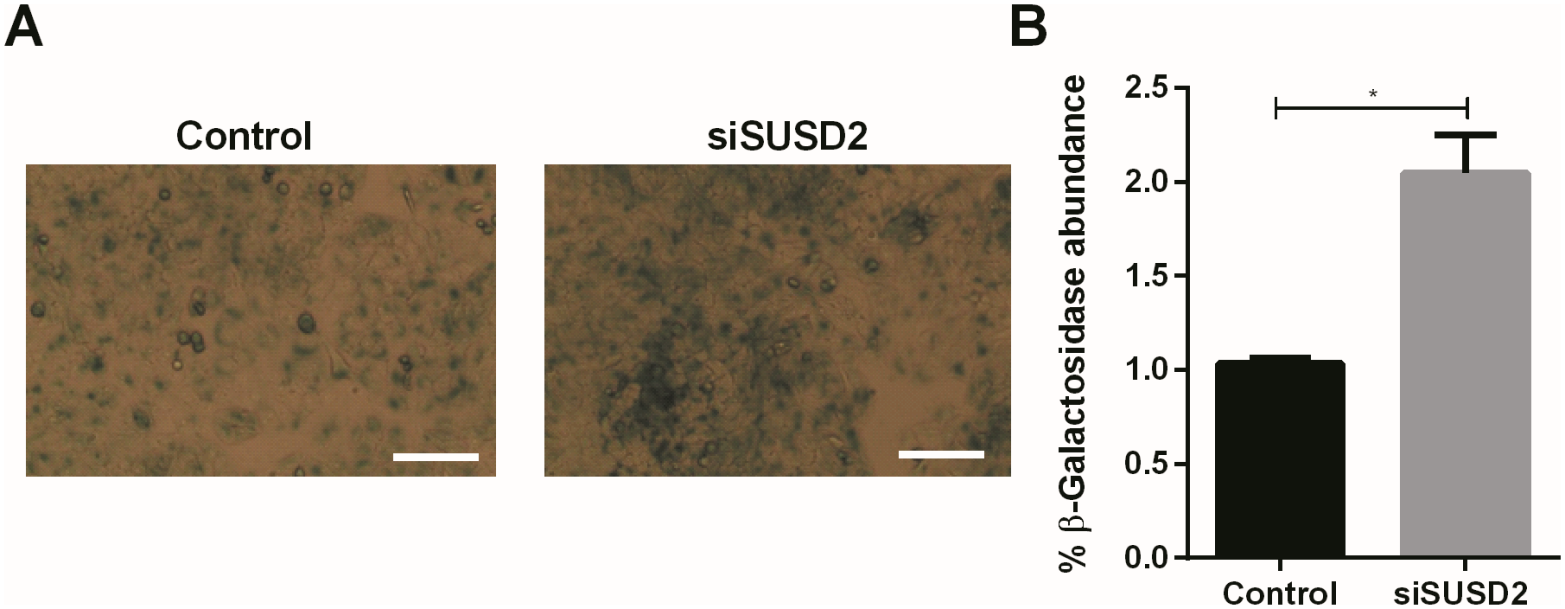
Influence of SUSD2 knockdown on cell senescence. (A) Representative pictures of Ishikawa cells stained for β-Galactosidase after SUSD2 knockdown. Scale bar 100μm. (B) Arithmetic means ± SEM (n = 3–8) of relative abundance of β-Galactosidase. *P< 0.05 indicates statistically significant difference from control cells using Student’s t-test.

To explore if loss of MSCs marker SUSD2 influences cellular senescence, we transfected cells with siRNA-SUSD2 or with Non-Targeting (Control) siRNA and measured senile associated β-Galactosidase (SAβ-Gal) activity. As shown in Fig 3A & 3B, siRNA-SUSD2 significantly increased blue staining indicative of SAβ-Gal activity and thus senescent cells.

**Fig 4:**
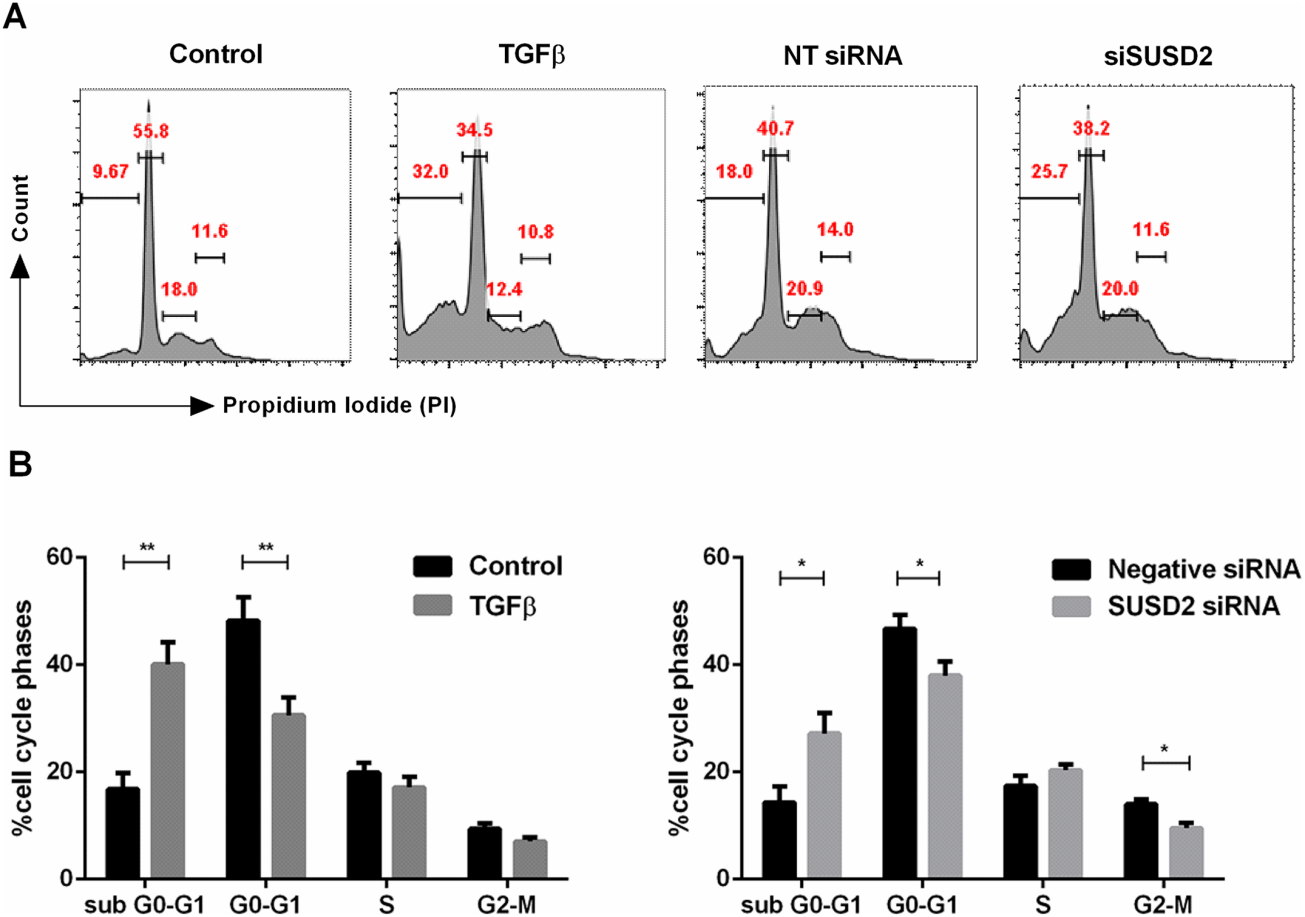
Effect of SUSD2 on cell death. (A) Representative FACS plots showing cell cycle progression of Ishikawa cells characterized by Propidium Iodide (PI) staining after 72h TGFβ (10 ng/ml) or Non-targeting (NT) or with SUSD2 siRNA treatment as indicated. Sub G0/G1 represents the apoptotic fraction. (B) Arithmetic means ± SEM (n = 5–6) of percentage cells in different cell cycle phases. *P < 0.05, **P < 0.01 indicates statistically significant difference from control cells using Student’s t-test.

We hypothesized that the senescence due to loss of SUSD2 could eventually lead to cell death. A final set of experiments was performed in order to define the fate of Ishikawa cells following loss of SUSD2. To this end, Ishikawa cells were transfected with NT or siRNA- SUSD2 or treated with 10 ng/ml TGFβ for 72h or left untreated. Cells were harvested and stained with Propidium Iodide. Cells were subjected to cell cycle analysis. A 72 hour treatment with TGFβ increased the proportion of sub G0/G1 cells indicating cell death. In keeping with these results, SUSD2 knockdown also profoundly compromised the viability of Ishikawa cells with a parallel increase of apoptotic cells (Fig 4A & 4B).

## Discussion

SUSD2 is a newly described endometrial mesenchymal stem cell marker and cytokine [7, 20, 21, 29, 34, 35]. Increasing evidence suggests that SUSD2 plays a key role in tumorigenesis [22, 27, 28, 36–38]. Two previous studies [37, 38] described the mouse homolog SUSD2 as a potential tumour suppressor. By contrast, Watson *et al*., [22] reported that SUSD2 was overexpressed in breast cancer, and indicated that SUSD2 enhanced the invasion of breast cancer cells. Further, this report showed that accelerated tumour formation and decreased survival rates in mice with tumours expressing SUSD2 were also observed in a syngeneic mouse model [22]. Our present observations confirm that SUSD2 is present in endometrial cancer cells [27] and that SUSD2 expression is sensitive to TGFβ signaling [7]. Our observations reveal that SUSD2 participates in the regulation of apoptosis and senescence in endometrial cancer cells. Treatment of endometrial carcinoma cells with TGFβ reduced the expression of SUSD2. This finding agrees with a previously published report that TGFβ plays a role in SUSD2+ stemness [7]. To mimic this negative effect of TGFβ we targeted SUSD2 using small interfering RNA (siRNA). We show that downregulation of SUSD2 increases apoptosis (sub G0/G1) and decreases G0/G1 arrest and cell senescence *via* SMAD2/3. However, another study in colon cancer has suggested that overexpression of SUSD2 alone or together with its ligand can inhibit colon cancer colony forming units [39]. Cell cycle arrest only happened at G0/G1 phase when SUSD2 and its ligand C10orf99 were present together, SUSD2 overexpression alone was not sufficient to induce G0/G1 phage cells arrest [39]. Data on the role of SUSD2 are conflicting and it is likely that SUSD2 regulates specific signal transduction processes determined by the cell type and by the state of cell differentiation or pathology.

Galectin-1, encoded by the *LGALS1* gene, is an interaction partner for SUSD2 [22]. Galectin-1 is expressed by the endometrial stromal cells throughout the menstrual cycle [40]. Galectin-1 has been extensively studied and is implicated in tumour transformation, cell cycle regulation, apoptosis, cell adhesion, migration and inflammation by modulating cell-cell and cell-matrix interactions [41]. Galectin-1 downregulation can inhibit invasion in prostate cancer [42]. We observed that Galectin-1 is reduced upon SUSD2 knockdown, but did not investigate the invasive capability. Moreover, the promoter of SUSD2 contains non-typical CpG islands, which can be restored by 5-azacytidine [39], indicating that promoter methylation manipulates its expression directly or transcription factors regulating its expression are epigenetically regulated. However, further studies are warranted to test this hypothesis in endometrial cancer cells.

In a liver cancer model, SMAD2/3 is upregulated in cells undergoing senescence [33], in keeping with our results. Oncogene-induced senescence (OIS) is a powerful way of tumour suppression [43–45]. Senescent cells implement a complex pro-inflammatory response termed the senescence-associated secretory phenotype (SASP) to eliminate premalignant cells [46]. The SASP reinforces senescence, activates immune surveillance and paradoxically also has pro-tumorigenic properties [47–49]. Whether loss of SUSD2 could activate the immune system remains to be determined. The SASP can potentiate the tumorigenic properties of cancer cells, recruit the immune system to eliminate premalignant cells [47, 50, 51] or reinforce senescence [33, 52].

In summary, we show that SUSD2 is present in endometrial carcinoma cells. We further show that suppression of SUSD2 expression following treatment with TGFβ or by siRNA increased apoptosis and senescence. Taken together these findings suggest that SUSD2 is a potential chemotherapeutic target in human endometrial cancer.

## Supporting information

S1 Fig Titration and Time-course of the TGFβ effect on *SUSD2+* Ishikawa cells. (A) Arithmetic means ± SEM (n = 3) of the fold change of SUSD2+ expressing Ishikawa cells. Bar graph showing the effect of increasing TGFβ concentrations (1, 5, 10, 20, 50 ng/ml) for 72h Data are depicted as fold induction relative to untreated (Control) samples. *P< 0.05, **P<0.01 indicate statistically significant difference from control cells using Student’s t-test. N.S- is non-significant. (B) Arithmetic means ± SEM (n = 3) of SUSD2+ (fold change) Ishikawa cells. Data are depicted as fold induction relative to untreated (Control) samples. *P< 0.05, **P<0.01 indicate statistically significant difference from control cells using Student’s t-test. (C) Arithmetic means ± SEM (n = 3) of the fold change of *SUSD2* mRNA transcripts in Ishikawa cells. Data are depicted as fold induction relative to untreated (Control) samples. *P< 0.05 indicates statistically significant difference from control cells using Student’s t-test.
(TIF)

S2 Fig Knockdown of SUSD2 decreased LGALS1 and MKi67 transcript levels in Ishikawa cells. (A) Arithmetic means ± SEM (n = 4) of *LGALS1* normalized to *L19* transcript levels in Ishikawa cells. (B) Arithmetic means ± SEM (n = 4) *MKi67* transcript levels normalized to *L19* transcript levels in Ishikawa cells. Data of both graphs are depicted as fold induction relative to transcript levels of vehicle samples. *P< 0.05, **P<0.01 indicate statistically significant difference from control cells using Student’s t-test.
(TIF)
