# Supplementary figures and images for: Downregulation of endometrial mesenchymal marker SUSD2 causes cell senescence and cell death in endometrial carcinoma cells
Source: PLoS One. 2017 Aug 25;12(8):e0183681. doi: 10.1371/journal.pone.0183681 (PMC5571916; doi:10.1371/journal.pone.0183681)

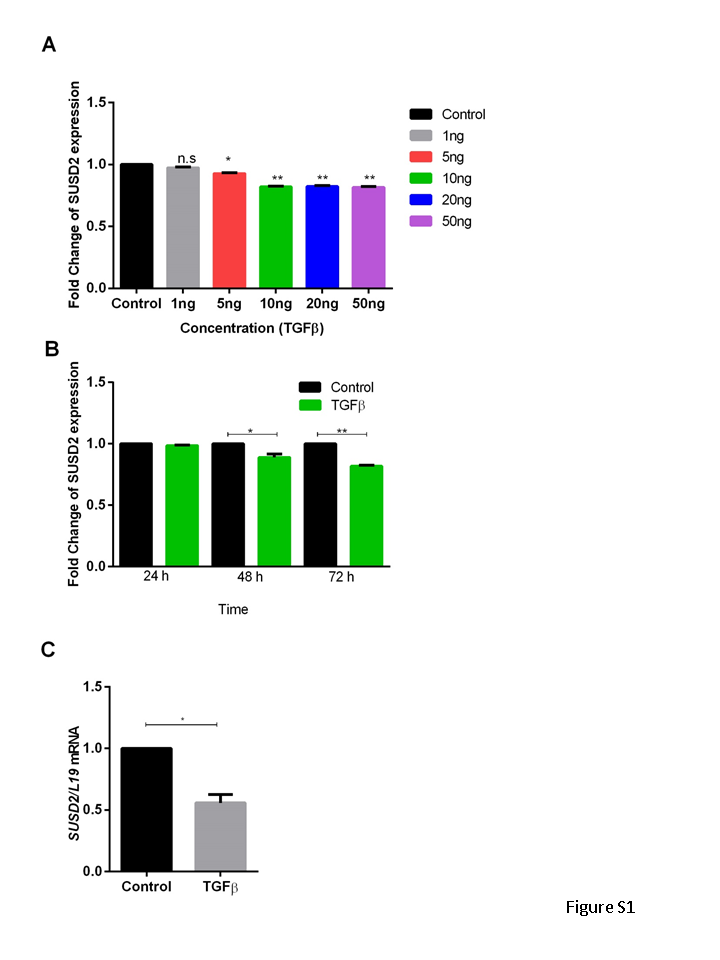

Supplement: S1 Fig — (A) Arithmetic means ± SEM (n = 3) of the fold change of SUSD2+ expressing Ishikawa cells. Bar graph showing the effect of increasing TGFβ concentrations (1, 5, 10, 20, 50 ng/ml) for 72h Data are depicted as fold induction relative to untreated (Control) samples. *P< 0.05, **P<0.01 indicate statistically significant difference from control cells using Student’s t-test. N.S- is non-significant. (B) Arithmetic means ± SEM (n = 3) of SUSD2+ (fold change) Ishikawa cells. Data are depicted as fold induction relative to untreated (Control) samples. *P< 0.05, **P<0.01 indicate statistically significant difference from control cells using Student’s t-test. (C) Arithmetic means ± SEM (n = 3) of the fold change of SUSD2 mRNA transcripts in Ishikawa cells. Data are depicted as fold induction relative to untreated (Control) samples. *P< 0.05 indicates statistically significant difference from control cells using Student’s t-test. (TIF) [file pone.0183681.s001.TIF]

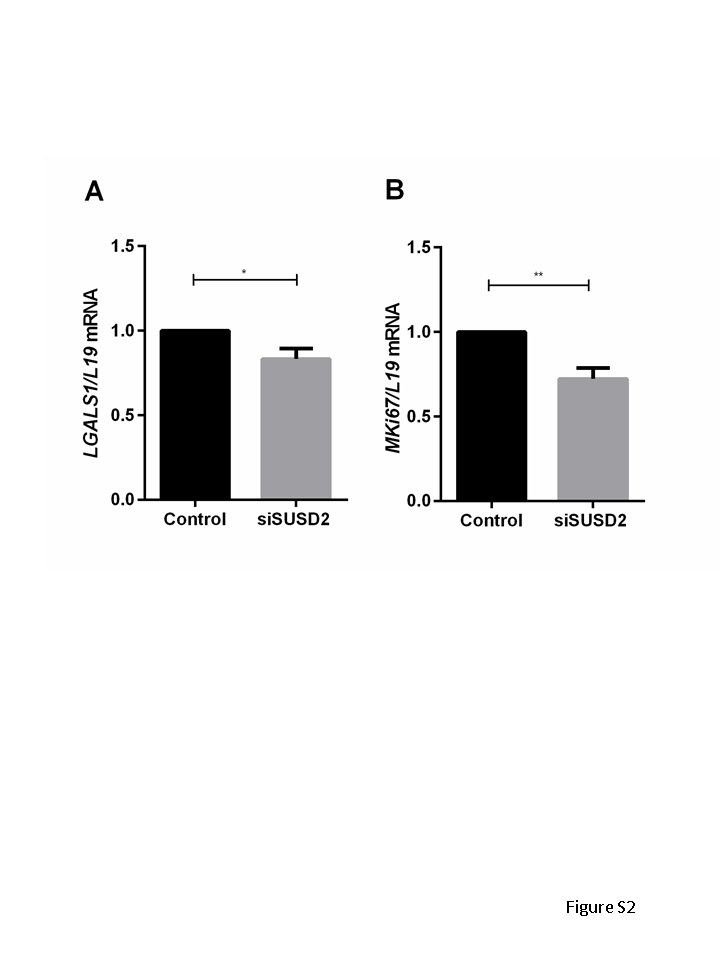

Supplement: S2 Fig — (A) Arithmetic means ± SEM (n = 4) of LGALS1 normalized to L19 transcript levels in Ishikawa cells. (B) Arithmetic means ± SEM (n = 4) MKi67 transcript levels normalized to L19 transcript levels in Ishikawa cells. Data of both graphs are depicted as fold induction relative to transcript levels of vehicle samples. *P< 0.05, **P<0.01 indicate statistically significant difference from control cells using Student’s t-test. (TIF) [file pone.0183681.s002.TIF]
